# Ovariotomy—Its Relations to Septicæmia, Pyæmia, Etc.

**Published:** 1874-08

**Authors:** J. T. Gilmore

**Affiliations:** Professor of Surgery in the Medical College of Alabama.; Mobile, Ala.


ATLANTA

MEDICAL AND SURGICAL JOURNAL.

VOL. XII.] AUGUST—1874. [NO. 5.

### Original Communications.

### OVARIOTOMY---ITS RELATIONS TO SEPTICÆMIA, PYÆMIA, &c.

BY J. T. GILMORE, M.D.,

PROFESSOR OF SURGERY IN THE MEDICAL COLLEGE OF ALABAMA.

*Read before the Mobile Medical Society.*

A colored woman, Jane Sloan, aged between forty and forty-five years, was sent me about the 1st of April, by my esteemed friend, Dr. H. Isard, of Garlandsville, Mississippi, to be operated on for ovarian tumor. The tumor was immense, the abdominal distension reaching a circumference of forty-five inches; and the tumor, from its large size and weight, had become somewhat pendu'ous, so that it hung between her thighs, reaching nearly to her knees. She stated that her health, previous to the appearance of the tumor, had been uninterrupted. She was the mother of three children, all of whom were still living, the youngest being now about ten years of age.

The first presentation of her trouble commenced some seven years ago, by pain, fixed mostly in the lower part of her abdomen and on the right side. For this she was blistered over the painful spot, and treated by internal remedies, without deriving any benefit whatever from the treatment. After several months of suffering in this way an enlargement showed itself in the hypogastric region, and, as she could best recollect, on the right side. This gradually enlarged, yet not interfering with her health or comfort, except mechanically, until it filled the abdomen, reaching to the ensiform cartilage.

Dr. Isard, who was her physician, drew off, by tapping, its contents, and diagnosticated the tumor as ovarian. The refilling was not rapid, another tapping not being necessary until two years ago. During this time the cyst had filled again, until it reached the dimensions already mentioned.

When she reached the city her catamenia was upon her, and the operation was delayed until this ceased. To make out a proper diagnosis I aspirated the tumor with a very small needle, and obtained a small quantity of the fluid for examination, which was examined both chemically and microscopically. The chemical examination showed the fluid to be neutral; heat gave a whitish precipitate, which was entirely cleared up by nitric acid. The specific gravity was 1010. The microscopical examination, made by myself and Drs. W. D. Bizzell and Rhett Goode, exhibited epithelial cells and the granular cell described by Dr. Thomas M. Drysdale, of Philadelphia; yet neither of these varieties of cells were at all abundant. There were seen some few blood corpuscles and oil globules. A physical examination of the abdomen found dullness, by percussion, at every point, with distinct fluctuation from every part of the distended abdomen.

The history of the case, and the examination thus far, pointed to a unilocular cyst of immense size, that came from the broad ligament of the right side. Was it a cyst of the broad ligament, or was it an ovarian cyst?

Dr. Atlee, in his published experience, seems to rely confidently upon the absence of albumen in the fluid to establish his diagnosis that the cyst is not ovarian. The researches of Dr. Drysdale, with the microscope, in the examination of fluids from abdominal tumors, gave grounds for the opinion that ours was a case of unilocular ovarian tumor.

The menstrual flow at this time prohibited an examination of the uterus. After waiting for several days, menstruation in the meantime having ceased, we examined the pelvic organs, and found the bladder and uterus both crowded down into the pelvis; the uterus lying below and behind the tumor, with its body pushed to the left side. The sound was not introduced into the cavity, thinking that the irritation thus created might damage the successful issue of an operation. The woman was considerably emaciated, as she said, entirely from the annoyance caused by the mechanical interference with the organs involved, mainly in nutrition.

At no time had she suffered from any indication of peritonitis. The irritation following the tapping had scarcely been sufficient to confine her to her bed. Reviewing her case with all its interesting points, it was agreed that the tumor was ovarian; that it was unilocular, and that in all probability it was non-adherent. Then again, considering the woman's condition, with every organ unimpaired, except so far as disturbed by the pressure and weight of the tumor, ovariotomy was decided upon, and the operation was set for Monday, the 6th of April, at one o'clock. On that day, in the presence of Drs. Ross, Anderson, Gaines, Owens, Reese, France, Sherrard and Smith, and with the assistance of Drs. Fournier—who administered the chloroform—Goode, Bizzell, and O'Donohue, I proceeded to operate. The patient yielded kindly to the anæsthetic, and when anæsthetized, the abdomen being held up by assistants, an incision about three inches in length was made in the median line, so as to strike the tendinous expansion of the external and internal and transversalis muscles above the point where it passes in front of the recti-abdominus muscles.

This I regard not an unimportant item in making the exploratory incision, for if the incision extends down into the preperitoneal space, the bladder, which seemingly rested low in the pelvis, might send a pouch into this space, and hence become endangered. I once witnessed an ovariotomy in which the bladder was pulled up, and by the merest accident escaped injury. Dividing layer after layer, the incision was deepened until the abdomen was opened. There seemed to be an entire absence of ascitic fluid, and before us was the glistening cyst. Then, by passing the finger down into the cavity towards the pubis, the incision was prolonged into the preperitoneal space, when it was found that the bladder was entirely out of the way. The next step of the operation was to demonstrate, beyond all peradventure, that the cavity was reached; the hand, after being dipped in warm water, was carried over the tumor until the umbilicus was passed. *En passant* I will remark, that this is an important item in operating, for in one of my cases such were the adhesions that I could not satisfy myself that I had reached the cavity until I extended the incision to the umbilicus, at which point the peritoneum is so closely cicatrized to the umbilical structures that it is inseparable, whereas the peritoneal investment of the tumor could be easily detected beyond that point. The next step was to tap the cyst, which I did with a large curved trocar, emptying it of two large bucketsfull of the fluid described. The buckets used were the ordinary commercial blue buckets. The assistants, in the meantime, keeping up pressure laterally and above the tumor, crowding it down towards the abdominal incision.

During the flow of the fluid a spell of vomiting came on, which dislodged the canula, but as I had taken the precaution to fix the cyst outside the opening in the abdomen, by grasping it with Musseus' forceps and dragging it out as far as I could, none of its contents escaped into the abdominal cavity. In consequence of this at least a gallon of fluid was lost. This will give an estimate of the size of the tumor, the cyst containing at least seven gallons of liquid. With its lessening it gradually yielded to the traction, and came out of the incision. An examination showed that it was the ovary of the left side converted into a unilocular cyst, over which extended the Fallopian tube. The pedicle of the tumor was broad, extending from the fundus of the uterus to the sigmoid-flexure of the colon. Atlee's clamp was applied to it and screwed as tightly down as possible, and in order to avoid leakage of blood, either from the stump above the clamp or from the cyst, Nott's rectilinear ecraseur was applied above the clamp, and the tumor was detached through the crushed tissues. The abdominal opening was then closed by six silk sutures. The patient, almost without shock, was returned to her bed, having been in the operating room only about a half an hour. Following the operation there was scarcely any noticeable constitutional depression or excitement. A many-tailed bandage had been applied over the abdomen, and the line of incision was protected by a layer of lint. The patient was ordered one grain of opium every six hours. Nothing worthy of mention occurred until the Monday morning following, one week lacking a few hours only. The patient was found nervous, with a sinking feeling and very manifest contractions of the muscles of the back and back of the neck. This stiffness gradually became tetanic, and by evening great difficulty of deglutition was experienced, and the patient wore the unmistakable tetanic physiognomy.

Dr. Fournier, the physician in charge of the hospital, who had a patient in the same ward convalescing from traumatic tetanus, put her upon the extract of the calabar bean. All to no purpose, however. The severity of the stiffness and the spasms constantly augmented until death put an end to her suffering, some sixty hours after the inception of the attack. As has been said by the inimitable Bowling, the result of this promising and brilliant case of ovariotomy may be set down as one of the "uncertainties of surgery."

This woman had brooked all the dangers that belong essentially and especially to the operation. Almost without an ache or a pain she emerged from the Scylla and Charybdis of shock, septicæmia, peritonitis, cellulitis, metastatic abscess or pyæmia, to die of a disease remotely connected with the operation. Peaslees' carefully and ably written monograph upon the subject of ovarian tumors, contains mention of but six cases from tetanus after this operation: one case operated upon by Barnet, one by Nelaton, and three in the practice of Spencer Wells. Regarding the mania that seizes hold of the professional mind when any innovation is introduced, and bears claims to confidence, subjecting almost every case of ovarian tumor to operation, it is strikingly remarkable how exempt this operation has been from fatality caused by this disease, considering the fact that it is almost the universal practice to deal with the pedicle by a process that peculiarly invites tetanic development. As was said of Nelaton's case by M. Des Chambre, that his patient "died cured," might be as well said of this case.

The incision had closed; the clamp was lying on the abdomen, held only by a little slough, and was removed without difficulty; the bowels had been opened by an enema, and the woman was begging to get out of bed when the disease first presented itself. Dr. Fournier opened the abdomen after her death, and found only a slight adhesive inflammation of the peritoneum along the course of the incision, and at one point the small intestine was adherent to the abdominal wall. The uterus was entirely free from marks of inflammatory trouble. There was a small fibroid situated on the posterior aspect of the body of the uterus, that had undergone calcareous transformation. There was no fluid of any character in the abdominal cavity, showing clearly and conclusively that the operation was a success, as far as an avoidance of the usual causes of death in such cases could make it.

In almost every paper gotten up by those who have practiced ovariotomy most extensively, it is claimed that a special fitness must belong to an operator before he is authorized to expect success in ovariotomy. The same might be said of every other surgical operation that endangers life. Every conscientious surgeon will bring to his aid not only the necessary anatomical knowledge to enable him to operate with precision, but he will feel himself acquainted with the nature of the complications likely to arise, by which his patient's life will be imperiled. It is true that many undertake, and operate successfully, for stone in the bladder, who are wholly ignorant of the anatomical structures cut through and the dangers likely to be engendered by the operation. They straggle into the bladder, and the recuperative powers of their patients come to their rescue; yet this does not justify such a person in doing the operation. McDowell knew nothing of the dangers of ovariotomy, save shock and peritonitis; yet his success was nearly up to those of modern renown. Then I claim that an experienced and careful surgeon, who operates with a fullness of knowledge and science, and who does not jeopardize organ or life, unless he knows that he possesses the necessary knowledge, is as competent to operate as those who count their operations by the hundreds. Ovariotomy belongs to the domain of surgery, and any competent surgeon can and is justifiable in undertaking it.

This last case is my ninth of ovariotomy, including one of normal ovariotomy, with three deaths. I have operated on all cases that have sought operative interference. The second of my fatal cases was a patient brought to me from the interior. She had been tapped a number of times at short intervals, the tumor refilling rapidly, and from these frequent tappings and the escape of fluid into the peritoneal cavity, a peritonitis was lighted up which had obliterated the cavity, causing adhesions at every point. When she arrived I expressed the opinion that an operation would give her scarcely a chance. She was so prostrated that she was unable to get out of bed; she was suffering from hectic; her appetite was gone, and without the operation she could not have possibly lived many days. The abdominal enlargement was considerable. I advised against any interference. I shall never forget the importunities of this poor woman, claiming that her chastity demanded it, and if life could only be spared her one hour after the operation her mission to me would be fulfilled, and that she would die with an unspeakable comfort in the satisfaction of having demonstrated to her slanderers her innocence of crime. I opened the abdomen and found the cyst closely adherent everywhere; the cyst wall was softened, and, when tapped, pus of an unhealthy character poured out. The adhesions were recent and were easily broken down. I found no difficulty in enucleating the sac, and ligated *en masse* the pedicle. When the operation was completed and the tumor was exhibited to her, with a heroism that only the sting of the wounded innocence of a true woman could call forth, she seemed to forget her dying condition, and to the Sister of Mercy who had charge of her after the operation, she unbosomed her tale of woe, and defended the operation in her case for the comfort it brought her in her dying moments. She survived the removal of the tumor about sixty hours, dying of exhaustion.

My other fatal case was one of dermoid cyst. This case, Mrs. C., of Clinton, Mississippi, was placed in my hands for treatment by Dr. Webb, of this city. She came to Mobile to visit her brother, and obtained advice. She was a badly nourished, delicate woman of twenty-seven years. She had been married for seven years and had never conceived. At the age of seventeen she had suffered from a severe attack of typhoid fever, that left her with a deranged digestion and menstruation, the catamenia recurring every two or three weeks irregularly. After her marriage she contracted a chronic intermittent, and about twelve months before coming to Mobile she had a severe attack of remittent bilious fever, that left her prostrate and with a constant burning pain in the right side.

The physician who attended her last attack of bilious fever, first found the tumor several months after her recovery from the fever. This gradually increased until it had attained the size of a large human head, situated in the lower part of the abdomen, with its largest development in the left iliac region. By manipulation an indistinct fluctuation could be elicited. The fluctuation could be most plainly elicited by palpation over the abdomen, with the fingers of the other hand in the vigina. The sound entered the uterus a normal depth, and showed that it was lateralized in its position to the left side beneath and in front of the tumor. With a large needle of Tieman's aspirator passed through the abdomen, I obtained a small quantity of the fluid, which flowed out sluggishly through the needle, finding it impossible to use the pump. It was of the consistence and color of rich, yellow butter, at about the temperature of blood heat. This, subjected to a microscopical examination, showed that it was an aggregation of epithelia in oil. This, of course, established the diagnosis, viz: that it was a dermoid cyst. The unpromising character of the patient for an operation was duly appreciated, yet her cachectic condition—having also rigors followed by fever and copious sweats daily, and that in despite of quinine, and the constant throbbing, burning pain in the right side, and, more or less, over the entire tumor—induced us to believe that the cyst was on the eve of suppuration, if suppurative action had not already been established in it. Believing this, interference of some sort was forced upon us. Nœggerath's method commended itself to us, and an attempt was made to empty the tumor by tapping through the vagina.

Dr. Webb and myself, after introducing Sims' speculum and locating a spot at which fluctuation could be recognized, with the patient in Sims' obstetrical position, concluded to plunge a large curved trocar through the vault of the vagina, and open the tumor and establish drainage through the vagina. This I did, but failed to get the fluid. The cause was easily explained afterwards, when abdominal ovariotomy was done. Failing to reach the fluid in this direction, we concluded to postpone further interference until she had recovered from what had been done. In about a week, at the Providence Infirmary, the patient having been carried there in the meantime, the abdomen was opened and the tumor enucleated. It was found to be attached to the right ovary, the ovary being outside the cyst, which laid as already described. After removing the cyst, it was found to contain a bunch of beautiful hair, as large as a woman's chignon, matted together. Into this, doubtless, the trocar had been plunged, and hence the failure to evacuate the fluid of the cyst. In addition to the chignon of hair, was found the blight of a lower jaw, with two well developed incisors. Also a considerable quantity of skin, from which grew scattering strands of hair, more than six inches in length. Several vessels required ligature, and when the abdominal opening was closed the patient was as comfortable as could have been expected under the circumstances. The shock, however, was prolonged; her depression increased, aad she gradually collapsed, and died in about twenty hours after the completion of the operation. I attributed her death to shock.

I have thus enumerated these three cases of fatal ovariotomy simply to open up a discussion of the causes that legitimately and essentially render ovariotomy a dangerous operation. The two last cases mentioned perished from shock. It was feared and believed that the operation gave them but small chances of relief before it was done. Yet every surgeon who feels his responsibility to his patient would have operated when the operation was sought, feeling, when his patient succumbed, that he had simply discharged an unpleasant duty. The shock was feared, and everything that we could think of to prevent its fatal effects was done.

I read with peculiar interest the views of Dr. J. Marion Sims on the subject of drainage, practiced to prevent septicæmia, immediately after ovarian operations, and I agree with him that many of the cases of death, usually ascribed to shock, legitimately belong to the influences of septic causes. In a discussion that took place between himself and Dr. Nœggerath, reported in the November number of the *American Journal of Obstetrics*, he strongly takes the ground that infinitesimal quantities of poisonous exudations in the abdominal cavity may be rapidly fatal. When we reflect that the peritoneum is largely endowed with absorbents, and before their activity of function is impaired by inflammatory invasion, poisonous substances coming in contact with the peritoneal surface, patients may succumb outright as though they were dying of shock. The very important question arises from this discussion, what is the earliest time at which septic causes can be brought into effect after fluid exudations escape into the abdominal cavity after an operation in which the abdomen is opened? When we reflect how rapidly post-mortem changes take place; indeed, when we recall the fact that rigor mortis supervenes upon death usually within twenty-four hours, thus showing that structural alterations have taken place in the muscular system, and how often poisoned wounds are obtained by post-mortem examinations of bodies in a few hours after death, it is not difficult to imagine how, in a few hours, fluids remaining in contact with the peritoneum might overwhelm the vital powers and render life extinct. Experience in dissection wounds abundantly show that putrefaction does not beget septic agents, but seems to lessen the danger of the poison. At this point we might be met with the query: How is it that oftentimes pus following peritonitis is retained in the cavity without rapidly producing death? This is easily understood by recollecting that the absorbing power of the peritoneum is well nigh, if not entirely, destroyed by an established attack of inflammation, and hence this septic element does not gain admission into the circulating fluids.

To discuss this subject of septicæmia properly is a vast undertaking. The phenomena from septic influences are almost as various as the physiological effects of different remedies, each septic cause producing its own and peculiar influences. Surgical fever, septicæmia, erysipelas, angeioleucitis, secondary hemorrhage, hospital gangrene, pyæmia, and, I believe, tetanus, are so closely linked together that a full knowledge of one cannot be had without a full understanding of the whole. The ordinary surgical fever bears to septicæmia the same relation that a febricula does to real fever; and the well known fact that surgical fever usually shows itself by the third day is a strong link in favor of the notion that septicæmia may be rapidly caused when the peritoneum has been assailed, for it is the cold stage of septicæmia that we are now considering, and not the reactionary condition evidenced by fever. If the septic agency of exuded fluids left in contact with the raw surfaces of an amputation, say of the thigh, can produce well marked depression, oftentimes becoming alarming, how much more to be dreaded would the same fluid be in contact with the absorbents of the abdominal peritoneum, as yet undisturbed by either inflammation or injury? What the intimate nature of septic poison is, neither microscopists nor chemists have been able to decide with certainty. That very variable thing called hospital air is oftentimes thought to have much to do with its origin; and that something that infests hospitals, that predisposes to the troubles mentioned as akin to septicæmia, has in vain been attempted to be explained by pathologists as due to living organisms existing in the atmosphere, especially in crowded hospitals.

I have said that the phenomena from septic influences are almost as various as the physiological effects of different remedies. Indeed, under the broad term of septicæmia we might include those influences that are the special dread of surgeons: erysipelas is of septic origin; secondary hemorrhage is of septic origin; hospital gangrene is of septic origin; angeioleucitis may be of septic origin; pyæmia and tetanus may be of septic origin; but that form of septicæmia that we are now considering is in no way connected with hospitalism. It can be equally well developed in the purest of mountain air, or in the marshy lowlands, or the crowded wards of a large hospital. The only safety that a patient derives from hygienic surroundings is, that the constitutional vigor is better sustained against the ravages of a most depressing poison, absorbed by the peritoneal absorbents from an exuded sanguinolent fluid in the peritoneal cavity, and the septic agent is probably less energetically generated.

But this poisonous element is as certainly created when the natural cause for it exists, as post-mortem decay follows death, and our only safeguard is in properly cleansing the cavity of all blood and establishing a proper drainage.

Pyæmia, as I shall define it, rarely plays a part as a sequel to ovariotomy. Nearly all authors and writers so confound the terms septicæmia and pyæmia as to make them synonymous. But I shall regard pyæmia as coming from thrombosis. Pus in the blood, is only generated by softening thrombi, and thrombosis, as we all know, originates most frequently in inflamed bones, especially when the medullary cavity is opened; and to illustrate this I have brought before you a specimen of osteomyelitis, invading the tibia and fibula following a severe fracture of both of these bones. The case I have still under my observation, and because of its peculiar interest in this connection I will give the details of it. Thomas Harrison, a lad aged eighteen years, fell on the last day of March from the top of the exposition hall through the hatchway, a distance of near forty feet, striking the sleepers of the building before the floor was laid. He received from the fall a fracture of the right clavicle, fracture of the left tibia at the junction of the middle and lower third, and a fracture of the middle and upper third of the fibula of the same leg. A splinter of considerable size passed through the outer part of the soft parts covering the orbit of the right eye just below the external commissure of the lids, traversed the ball and forced the lens out of its bed, leaving it just beneath the conjunctiva on the inner aspect of the globe. A large internal iridectomy was also made by the splinter. In addition to these injuries the shock was fearful. Eight months previous to this accident he received a fracture of the tibia at the same point by a tumble down the custom house steps, caused by an epileptic convulsion, to which he is subject. This fracture was treated by the plaster-paris bandage, and a speedy union was obtained.

When I arrived at the site of the recent accident I found the boy almost lifeless. I placed his leg in Neil's fracture box and had him carried to his father's house. The shock was fearfully prolonged. For two weeks I would not have been surprised to have found crape on the door on my arrival at each visit. From this condition, however, he slowly emerged only to encounter a severe attack of pneumonia, which possibly came from the bruising of the right side of the chest, although I could not find any broken rib or ribs. As soon as his condition would admit of it I re-arranged the dressing, making the necessary extension and counter extension by adhesive straps, hoping that union would speedily ensue. In this, however, I was disappointed. The foot soon commenced to swell and the leg became painful, and a small slough showed itself on the posterior aspect of the leg, which necessitated the suspension of the limb in Smith's wire splint. In this position it was kept, and seemingly improved for a couple of weeks, the slough mentioned having in the meantime separated, emitting a considerable quantity of pus, which evidently came from the diseased bone. With a probe I readily recognized denudation of both the tibia and fibula at the points of fracture. The boy's condition now being good, I hoped to get under my control the inflammatory action, and when the proper time arrived to remove the diseased bone and ultimately secure union. But in this, I was baffled. The boy was suddenly seized with a horrible rigor, that prostrated him. Upon my arrival, in some two hours, I found him almost pulseless, with skin cold, and vomiting. He complained of pain at the groin of his broken limb, and there, in the saphenous opening, I found a thrombus, seemingly blocking up the long saphenous.

The next day, his depressed condition persisting, I called in my able friends and colleagues, Drs. Gaines and Fournier, and discussed the propriety of an amputation, even in his depressed condition. Here we had thrombosis — not pyæmia as yet— pyæmia was the danger to be dreaded. Here was a patient in private practice, remote from hospital surroundings, that would, in my judgement, certainly have rapidly succumbed to pyæmia but for the timely removal of the diseased limb. It is true that thrombosis does not necessarily imply pyæmia, but let thrombosis occur in a patient broken down from any cause, and his dangers of pyæmia are very great. The case now under consideration was powerfully depressed by the thrombosed action.

The leg became, in twenty-four hours after this complication set in, tender, much swollen, and was covered with an erysipelatous blush. The skin covering the thrombus at the saphenous opening wore a red, livid appearance. Now, in this condition, with a septic element seemingly at work, a condition at the sites of fracture and thrombus that no surgeon would have hesitated to have pronounced erysipelas, amputation was done, and the boy's present condition shows that the operation was wisely counseled. The amputation was done at the knee joint; for, as you see by the specimen, the whole of the tibia was involved more or less in disease, and in the lower fragment you observe the softened and porous condition; and the medullary cavity of this fragment was actually filled with pus. I show you also a section of the long saphenous and posterior tibial veins made by my student, Mr. Harrison. As you observe, both of them are blocked by thrombi. In the saphenous the thrombus is organized; in the posterior tibial it is not as yet, and may be post-mortem. As soon as the amputation was done and the effects of the chloroform passed off, the boy reacted, and in an hour's time his condition was infinitely better than it had been for the three previous days. The outbreak of the erysipelas, I think, was due to the thrombosis, and in many instances in which we have pyæmia accompanying erysipelas and angeioleucitis, I am confident that the erysipelas and angeioleucitis are caused by the obstructed venous return, having its origin in a thrombus, or thrombi. In this case I think that the prevailing septic agency, that has invaded every part of our city and caused erysipelas in nearly every one who had an open wound, played a part; but when the patient's system was rid of its greatest source of irritation, his recuperative powers came to his rescue and saved him from a full attack of erysipelas of the leg, and death from pyæmia. The thrombus in the groin has become organized and is rapidly disappearing, and had it not been in our power to relieve him of the producing cause of his thrombosis and depression, and of his erysipelas too, I think his thrombi would have softened into pus, and it, with detached thrombi, would have been let loose in the general circulation, developing multiple abscesses, with accompanying rigors, succeeding sweats, exhaustion and death. Doubtless Dr. Ketchum recollects a case very much analogous, that occurred in our experience some two years ago; and Dr. France was with me also in a similar case. In both of these cases amputation was done in the lower third of the femur with the result of saving the lives of both patients. I have, in a number of cases, under similar circumstances, operated with results that encourage me to repeat the effort to ward off the outbreak of pyæmia under circumstances apparently the most desperate.

In the May number of the *London Lancet* I find an interesting discussion upon the subject of pyæmia, as occurring in private practice. That such an event can occur is too well evidenced to my mind to admit of a doubt. Drs. Gaines and Ketchum doubtless recollect well the case of Mr. Wash Lott, whom we attended last summer. Mr. Lott was first taken with what was believed to be the passage of a gall stone, which caused him intense suffering for several days, which was succeeded, seemingly, by hepatitis and gastro-enteritis. For this he was treated by Drs. Gaines and Ketchum, and when apparently convalescing, he was suddenly seized with a chill, that greatly prostrated him, and this, in turn, was succeeded by a fever and the characteristic hot skin and copious sweating of pyæmia. This attack came on in about two weeks after the hepatitis and gastro-enteritis were inaugurated. This chill was followed by a succession of similar chills, recurring without any regularity. But he seemed to commence again to convalesce, and the improvement lasted about two weeks, when again a repetition of the irregular chills, with the before-mentioned hot skin and sweats, were inaugurated. It was in the last attack that I saw him with Dr. Gaines, who had fully made out his case as one of abscess of the liver. These symptoms continued until he died, worn out by a constantly recurring attack of chills, fever, and exhausting sweats. He had, as you would suppose, the jaundiced hue, earthy skin, pinched face, and shriveled neck, that belongs to metastatic abscess. A post-mortem cleared up the whole case, and sustained in every particular the diagnosis. In the gall bladder were found some twenty-five or thirty small gall stones. The liver was much enlarged, and soft and friable at many points. The vena porta was literally blocked with thrombi, and, mainly in the left lobe of the liver, were found many points of softened thrombi, with more or less intermixture of pus.

This case is not a novel one, but is brought in here because it beautifully illustrates how thrombi soften and degenerate into abscesses, and kill patients precisely as thrombi may do when formed in other localities and from other causes. In Lott's case we had multiple abscess, or pyæmia, if you choose to so call it, occurring without a wound, without previous suppuration, and without any septic agent that could possibly exert any influence, unless we admit malaria among the septic agents. The solution of the thrombosis is easy. He had, in the outset, the passage of a gall stone, which lighted up, more or less, hepatitis and gastro-enteritis, and the thrombosis of the portal vein was induced possibly by contiguity to the ductus communis coledochus. This thrombosis occurring in a patient more or less depressed by malarial influences, and also in a locality where it greatly embarrassed the proper discharge of functions of an important organ, suppuration and death were the end.

Pyæmia from thrombosis is fully illustrated in the parturient woman. After every confinement the large sinuses of the gravid uterus become filled with thrombi, and when everything goes well these thrombi become organized, undergo fatty degeneration, and are absorbed, thus obliterating the sinuses by the time the organ has completed the process of involution. Now, suppose a lying-in woman is depressed from any cause, either physical, emotional, or by some septic agent; she may succumb to pyæmia from a softening and detachment of the uterine thrombi, developing pyæmia.

We have now instanced how pyæmia may be the offspring of osteo-myelitis; we could also mention how erysipelas and angeioleucitis might cause it. We have also mentioned a case of pyæmia following thrombosis of the portal vein, and cursorily referred to pyæmia in the lying-in woman, which is unscientifically known as puerperal fever; and I now call your attention to that connecting link between pyæmia and septicæmia, which, as yet, has not been assigned its proper place in surgical nomenclature. When it occurs in crowded hospitals, as it most frequently does, it is called hospitalism. When it occurs in chronic abscesses it is indiscriminately called septicaemia or pyaemia; and when it is the sequel to urethral fever, or a poisoned wound, it is thus loosely designated. Now, this condition may complicate pyæmia as I have described it; indeed, it oftentimes does, but is witnessed frequently independently of thrombosis; and to illustrate what I wish, I will call to your attention a young man, a private in the 18th Mississippi Regiment, whose arm was amputated by Dr. J. M. Holloway, now of Louisville, Kentucky, then surgeon of this regiment, for a wound received at the battle of

Ball's Bluff. I have seen many such cases, but instance this one because it was the first I ever saw of such cases, and it impressed me. The amputation was done by the flap method for a comminution of the bones of the forearm and elbow, caused by a wound inflicted by a minnie ball. He was treated in a hospital temporarily created in the lower story of a brick church, with some fifty others. Some two weeks after the amputation he commenced suffering with pain at the end of the stump. The wound had healed to a small opening, that was kept open by a ligature on the bronchial artery. With this pain, that increased toward night, more or less fever recurred with each exacerbation toward evening. The tongue became red and the mouth dry; the mind grew apathetic, or, in a word, all those symptoms that are known as typhoid, supervened. This lasted for about a week or ten days, when something like a rheumatic arthritis of the elbow of the opposite arm presented itself; in a few days the knee joints, and thus joint after joint became involved, until nearly every joint in the whole system was invaded. This inflammatory action, in forty-eight hours, as a rule, filled the articulation with a thin fluid pus. With his articular system thus burthened with pus, and his vital forces taxed beyond his powers of endurance, he died.

Now, this man had no symptom of thrombosis. There was no lung complication. The spleen, doubtless, was more or less enlarged, which is to be expected in all serious blood-poisonings. Yet his disease was as distinct from pyæmia, as we understand it, as scarlet fever is from yellow fever. This secondary surgical fever was quite likely brought about by an attack of osteomyelitis; but the septic influence of pus, the emanations from suppurating wounds, caused an atmosphere that favored, in all who were wounded, a phlogogonous action, that shows a preference for joints, but may develop abscess in other parts of the body.

Now, I never saw the test made, yet I confidently believe that if a lying-in woman was subjected to such an atmosphere she could scarcely escape pyæmia from the softening and detachment of the thrombi in the uterine sinuses. This condition usually occurs in those suffering from wounds, and those who are suffering from severe ones, especially if bone is involved, are more impressible than those who have simply flesh wounds. This form of septicæmia we might call hospitalism; for generally an atmosphere impregnated with emanations from pus creates it.

The opening of a chronic abscess is greatly dreaded on account of the rapidly fatal effects of septicæmia, produced by the atmosphere gaining access to the cavity of the abscess. Here a chemical change is wrought, that rapidly prostrates the energies and endangers life. In the present state of our knowledge it would be idle to attempt to state precisely what these changes are, and we can only deal with the facts as they exist.

This septicæmia that we see following the opening of chronic abscesses is, in my judgment, of the same character as that we observe in ovariotomy following the exudation into the abdominal cavity that succeeds an attack of peritonitis. Here the absorbents have been more or less impaired in their activity, and do not readily appropriate septic fluids, and death does not ensue so rapidly as in the primary form of septicæmia. We might multiply *ad infinitum* the varieties of septicæmia. Indeed, all atmospheric, telluric or contagious influences that produce disease might be brought under the general designation of septicæmia; but in surgical nomenclature we confine the expression to those poisons that are known to be generated within the system from its own secretions and excretions. Oftentimes, in persons broken down by long-continued genito-urinary irritation, we find a condition apparently precisely the same as that described as hospitalism, following urethral fever. Here we have a septic poison generated in the urinary secretion that finds its way into the system through a lacerated or abraded mucous membrane of the urethra, caused by the rude handling of an instrument, which takes place with the first passage of urine after the instrumentation, and a phlogogonous action is established that causes precisely the same phenomenon as that witnessed from the emanations of purulent secretions. Now, both poisons are analagous in pathalogical phenomena, and, if we could identify them, it is quite probable that we would find them the same, both being products of animal excretions.

The septicæmia of the puerperal state is well understood. Here we have possibly blood remaining in the uterus, or in the vagina, that develops different grades of the disease, depending in part on the susceptibility of the woman and the energy of the cause. So with the effusion of a peritonitis following confinement and pelvic cellulitis, that may end in chronic abscess, with all the dangers of chronic abscess elsewhere. Indeed, whoever understands thrombosis, pyæmia and septicæmia, and duly appreciates the causes of the latter understands the pathology of those diseases that are improperly classed under the one common head of puerperal fever. I will mention to you a case of curious interest that was brought before the Society last summer. It was the case of a young man about twenty-five years old, who, in a playful tumble with some of his comrades of a fire company, got a bruise of the scrotum, which put him to bed. The parts became so swollen and tender that Dr. Sherrard ordered them leeched. The day after the leeching he was taken with a shivering, followed by fever. When I saw him in consultation with Dr. Sherrard, some ten days after the leeching, his condition was emphatically that of septicæmia. There was no abscess about the scrotum, but simply the remains of an attack of erysipelas. He was having considerable fever every afternoon, with incomplete remissions in the morning. He also had a dry, red tongue and sore throat, that was evidently irritated by the swollen condition of the thyroid body, which had participated in the phlegmonous inflammation and run on to suppuration. Dr. Ketchum afterwards came in charge of the patient, and recognized the existence of the influence of a septic agent. There was a feature in this case worthy of mention. When the throat was irritated, there exuded upon its surface a thin, grayish pellicle, unlike diptheria in not being so exuberant, and the base, from which it might be scraped, still retaining its epithelium. This patient had abscess in various parts of the body: first, in the neighborhood of the knee-joints, then in his thyroid body, and, if I mistake not, along the course of the absorbents of the inner aspects of both thighs. These were preceded by streaks of angeioleucitis. Here was a case of septicæmia outside of the influence of anything like hospitalism. I think Dr. Sherrard's explanation of its origin is the correct one. He ascribes the septicæmia to the influence of a poisoned leech bite. He believes that the leecher employed leeches that had been used before on some other person, and in this way a septic poison was introduced that came near destroying the young man's life. For it was only after long suffering and the most careful attention and nursing with a change of air that he barely escaped with his life. This is the only case I ever saw disconnected with suppuration or urethral fever. It is interesting and curiously suggestive to study this subject in connection with irritative fever as witnessed in the latter stages of pulmonary phthisis, and chronic suppuration. But we have said enough for our purposes in studying ovariotomy.

Then to formulate and define pyæmia, as we understand it, it is metastatic abscess from softened and detached thrombi. Hospitalism is a septicæmia caused by emanations from suppurating wounds which attacks only wounded persons. Septicæmia may be either immediate or remote. Immediate, when caused by the re-absorption of fluids; remote, when due to an influence that begets that phlogogonous action described as hospitalism. Septicæmia may complicate pyæmia; indeed, it may be the producing cause of pyæmia, but is entirely a different pathological condition. When a surgeon is called to treat a patient requiring a surgical operation, he surveys the whole field of probabilities. In contemplating ovariotomy he discusses in his mind the powers that a patient can oppose to the dangers, first of shock, and secondly of immediate septicæmia, then peritonitis and pelvic cellulitis; of course there is some danger of remote septicæmia, pyæmia, erysipelas and tetanus, but they deserve no more thought at his hands than in other serious operations. Knowing the indications, he prepares himself to ward off these complications, if possible, and to control them if he finds it impossible to prevent them. He prevents shock by operating as rapidly as the safety of his patient will allow and carefully controlling all hemorrhage, having, if thought necessary, prepared her for the anæsthetic, by giving her a good slug of brandy or some other stimulant previous to its administration; taking care, during the operation, that she is properly guarded against cold. Against immediate septicæmia he protects her by properly securing all bleeding points, and thoroughly cleansing the cavity of all fluids before closing the abdominal section, and then by establishing, by whatever plan may commend itself to his judgment, a drainage that will give an outlet to any fluid that may be exuded from the stump or from adhesions, or from whatever peritonitis may be created, more or less of which is unavoidable; and the method suggested by Dr. Marion Sims certainly commends itself to my judgment as being superior to all other methods proposed. If a safe and reliable drainage could be established so as to keep the abdominal cavity free from all fluids, both sanguinolent and purulent in operations necessitating the opening of the abdominal cavity, the proper treatment of the pedicle would be a question of easy solution. I regret that I did not follow my instincts in the case of Jane Sloan, and deal with the pedicle in her case as I had done on former occasions, as I witnessed in two of Dr. Cochran's cases, and tie each vessel separately and make the puncture through the recto-uterine space as advocated by Dr. Sims. Had I done this I am quite sure that I could have added another to my successful cases of ovariotomy. It is true that there was prevailing at that time a peculiar condition of the atmosphere predisposing to erysipelas and tetanus; for, including this case, in the brief space of about three weeks, I encountered three cases of traumatic tetanus. Indeed, it seemed due to an epidemic influence, for the Registrar's report ending the week, April 18th, numbered fourteen deaths, and more than half were among children from trismus and convulsions. At this time there appeared an epidemic influence of erysipelas, and I had a patient to perish from secondary hemorrhage in a week after the cases of tetanus, following the ligation of the carotid for true aneurism, on the tenth day after the operation; thus exhibiting a high type of atmospheric influence that rendered all surgical operations peculiarly hazardous during its prevalence. Since the prevalence of dengue up to within the last month, erysipelas has been cropping out in every part of the city where I had a surgical case with an open wound; and in the city hospital after the death of Jane Sloan from tetanus, there broke out in quite a number of patients, with old ulcers of the skin, erysipelas.

The link that connects all those affections in the same class, which to the surgeon are his greatest source of alarm, viz: septicæmia, pyæmia, erysipelas, hospital gangrene, secondary hemorrhage, and tetanus, is an interesting study.

Jane Sloan would, I think, have resisted the influence had it not been for the bruising of the pedicle by the clamp, and her surroundings.

The advantages of drainage are well illustrated by the experience of the late Dr. Warren Stone, of New Orleans, in chest wounds. Dr. Stone observed that wounds of the pleural cavity, inflicted by large cutting instruments, were far less dangerous than those inflicted by smaller instruments, leaving simply a wound. At the time I heard his lectures on this subject, but little was known of septicæmia, yet he had observed that a free outlet relieved the system of irritative fever that the retention of blood caused, and that the parts locally involved were less likely to take on suppurative action, and end in chronic abscess or empyæma. Now, this clinical observation applies with more truth to the abdominal cavity; for peritonitis is far more fatal than pleurisy, and septic influences are more readily engendered. Then all this reasoning leads us to the conclusion that a proper drainage is the most important desideratum in the operation of ovariotomy. The clamp is inapplicable in many cases, and even in those cases best suited for its application it increases the dangers of tetanus and cellulitis, and oftentimes shock, by its dragging action on the pelvic organs. The next danger is peritonitis. Here our most reliable prophylaxis and remedy is opium. When the patient bears this remedy well, peritonitis is not usually a formidable complication. Then, finally, shock provided against, septicæmia prevented by drainage, and peritonitis warded off by the judicious administration of opium, ovariotomy, in suitable cases, is a legitimate surgical resource, and can be confidently undertaken by any competent surgeon.

The case of Jane Sloan causes me to repeat what is insisted upon by every experienced ovariotomist, viz: that ovariotomy should never be done in a general hospital, and that patients subjected to this operation should never be surrounded by other patients. If this woman had been in private practice and watched by a special nurse, her chances would have been increased tenfold. The crushed character of the wound of the pedicle and the peculiar condition of the atmosphere—the warm days and unusually cold nights—brought on an attack of tetanus, which special nursing and the quietude of separation from other patients might have prevented.

