# Errata

**Published:** 1874-07

**Authors:** 


### ERRATA.

(Please enter the corrections with pencil.)

Page 256, line 21—For recti-abdominus read recti-abdominis.
Page 260, line 29—For operatiou read operation.
Page 261, line 14—For Peaslees' read Peaslee's.
Page 261, line 16—For Barnet read Boinet.
Page 263, line 39—For Tieman's asperator read Tiemann's aspirator.
Page 272, line 10—For bronchial read brachial.

